# Evaluating the effects of environmental management practices on environmental and financial performance of firms in Malaysia: the mediating role of ESG disclosure

**DOI:** 10.1016/j.heliyon.2022.e12486

**Published:** 2022-12-21

**Authors:** Qaisar Ali, Asma Salman, Shazia Parveen

**Affiliations:** a Faculty of Islamic Economics and Finance (FEKIM), Universiti Islam Sultan Sharif Ali, Brunei Darussalam; b College of Business Administration, American University in the Emirates, United Arab Emirates; c National College of Business Administration and Economics, Lahore, Pakistan; d Association of Professional Researchers and Academicians, United Kingdom

**Keywords:** Environmental management, Environmental practices, Environmental performance, Financial performance, ESG

## Abstract

The concepts of environmental and business sustainability are well-established in the business lexicon of progressive firms. However, firms are yet to examine the effects of environmental management practices (EMPs) on environmental performance (EP) and financial performance (FP) by connecting the missing linkage of environment, social, and governance disclosure (ESGD). This study analyses the impact of EMPs on EP and FP and offers empirical evidence of whether ESGD mediates the relationship between EP and FP of firms in Malaysia. The data from 141 listed firms on Bursa Malaysia was extracted between 2009–2020. The data was analyzed using data envelopment analysis (DEA) and the generalized method of moments (GMM) technique. The findings reflect that EMPs have a significant positive effect on EP and all five proxies of EP have a significant positive effect on ESGD (except ‘S’) and FP. Also, ESGD together with its three proxies mediates the relationship between EP and FP. The findings of this research offer an empirical rationale for regulators and policymakers of industrial firms to accelerate their EMPs and improve ESGD mechanisms for better environmental and financial outcomes.

## 1 Introduction

Global businesses have started employing different environmental management practices (EMPs) to mitigate the effects of surging environmental threats (Tene et al., 2021). The findings of recent studies have confirmed that industrial firms play a concomitant role in the destruction of the ecological system and regulators, business managers, and scholars are exploring various methods to combat these issues (Ali et al., 2022; Pinto et al., 2018; Shahab et al., 2020). The current global business environment is complex which has further enhanced the uncertainties about gaining a competitive advantage and survival without complying with environmental legitimacy and addressing the concerns of stakeholders (Shahzad et al., 2020).

Simultaneously, scholars have suggested that strategic implementation of EMPs improves firms' environmental performance (EP) which alternatively contributes to improving the environment, social, and governance (ESG) and financial performance (FP) (Chen et al., 2018; Zheng et al., 2020). Proactive implementation of EMPs involves, developing a management system by synergizing organizational structure, planning environmental initiatives, sharing responsibilities, developing processes, and acquiring resources to implement, achieve, review and maintain an effective environmental policy (Aslam et al., 2021). The evidence of theoretical studies predicted that organizations’ voluntary commitment to environmental initiatives helps in gaining a competitive advantage by leveraging strategic resources (Zhang and Ma, 2021) and seeking a wider social acceptance by legitimatizing environmentally-driven operations (Elzinga et al., 2020). According to resource-based view (RBV) and stakeholder legitimacy theories, consistent organizational commitment of good EMPs may improve their EP which improves ESG and may result in financial benefits, competitive advantage, improved reputation, and positive brand image. RBV and institutional theories under similar settings further argued that the organizational commitment of good EMPs cajoles managers to develop environment-friendly initiatives which lead to better EP, ESGD, and improved FP (Feng and Wang, 2016; Hart, 1995; DiMaggio and Powell, 1983). Additionally, commitment to good EMPs leads to developing and maintaining an effective business connection with stakeholders and exploiting critical resources. Based on the proceeding argument, this study aims to investigate whether EMPs improve the EP and ESGD of industrial firms and alternatively affect FP.

Progressive organizations use environmental, social, and governance disclosure (ESGD) as a tool of their wider corporate social responsibility (CSR) to engage stakeholders and disclose their environmental policies, initiatives, and performance (Crane et al., 2019). Often, organizations are rated based on their good EMPs and EP which considerably contribute to strategic positioning and influence organizational perception, value, and image (Martins, 2005). Investors, for example, require ESGD information to assess the firm's ethical and sustainable performance for a better understanding and evaluation of the decision-making pertaining to ESGD issues (Amel-Zadeh and Serafeim, 2018; van Duuren et al., 2016). Therefore, ESGD is significant to scrutinize organizations' discrete claims about the contribution of information-based governance to CSR as well as examining strategic operational changes through EMPs (Depoers et al., 2016). Lastly, scrutinizing organizational response offer contemporary information on the implications of sustainability concepts in response to growing capital which will help in analyzing the investment and expenditure on ESG initiatives. This leads us to examine whether ESGD improves FP by mediating the relationship between EP and FP.

The past studies examining the relationship between EMPs, EP, FP, and ESGD have several limitations. According to the studies of Dragomir (2018), Henri and Journeault (2008), Trumpp et al. (2015), and Xie and Hayase (2007), organizations characterize environmentalism as a multilayer construct that determines two distinct characters i.e., EMPs and EP which are difficult to interrelate. Most empirical studies have focused on analyzing environmental approaches through the lens of economic benefits which primarily concentrate on the financial benefits of going green (Clarkson et al., 2011; Jiang et al., 2018; Jyoti and Khanna, 2021). Also, past studies instead of directly measuring the environmental initiatives of organizations have used environmental disclosure proxies which may render errors in capturing the actual EMPs and EP (Albertini, 2013; Deegan, 2013, 2017). Hence, it is argued here that the generalizing capacity of these studies is minimal. A few studies have attempted to develop scales to analyze EMPs (Al-Tuwaijri et al., 2004; Clarkson et al., 2008; Ilinitch et al., 1998; Montabon et al., 2007; Xie and Hayase, 2007) however, the failure to validate these scales raise concerns about the authenticity of these studies (Trumpp et al., 2015). Scholars have addressed these limitations by developing an exclusive EMPs scale using five sub-dimensions to statistically measure EMPs (Xie and Hayase, 2007). The statistical confirmation of this scale was validated by Trumpp et al. (2015) while measuring organizational EMPs. There is no evidence of a mutual agreement in the extant literature about the measurement of EMPs (Song et al., 2018). The emergence and discovery of new sources of information potentially contribute to the actual quality of EMP measurement (Hassan and Romilly, 2018; Tadros and Magnan, 2019) which indicates that the efficiency of EMPs, EP, and FP should be measured by using data envelopment analysis (DEA) (Aslam et al., 2021). Some emerging studies have corroborated the efficiency, EMPs, and EP of firms using DAE however, these studies were restricted to micro-level analysis (Jin et al., 2014; Wojcik et al., 2019; Zhou et al., 2006, 2007). Nonetheless, promulgated by the rare usage of DEA to measure environmental and financial efficiency at the organizational level, this study rendered the DEA approach to estimate the environmental (EMPs, EP, and ESGD) and financial performance of firms.

The social, environmental, and regulatory dynamics in Malaysia are considered relevant drivers to conduct this study. Recently, Malaysians were characterized as an environmental nation (Mei et al., 2016). Malaysia is touted as one of the developing countries in Southeast Asia and a pioneer of industrialization in the region through its economic growth. Industrialization has played a major role in expanding economic growth and facilitating the achievement of Mission (Wawasan) 2020 (Chin et al., 2019). However, in the process of industrialization and economic expansion, industrial firms have heavily polluted the environment (Awang et al., 2000) such as industries in Klang Valley, Penang and Iskandar contribute to one-third of the overall pollution of the country (Azmi et al., 2010). To tackle these issues, the government of Malaysia has imposed stringent environmental regulations on industrial firms.^1^ However, the effectiveness and compliance with these regulations by the firms through their EMPs and the interaction of these EMPs with the performance of firms need further investigation.

Based on the contemporary limitations of the prior studies and the relevance of current research to the Malaysian context, we seek to contribute to the extant literature in several ways. First, the present study empirically examines the interrelationship between EMPs, EP, ESGD, and FP which is expected to provide a benchmark for future research in similar settings (Chen et al., 2016, 2018). Secondly, the current research measures EP through major inputs and outputs of EP which substantially differentiates it from the past studies that have separately used the indicators of carbon emission (CO2), energy, waste, and water to measure EP (Arena et al., 2003; Clarkson et al., 2011). The present study employed major indicators of energy (input), sales (good output), and carbon emission (bad output) as the proxies of EP which represent the inclusiveness of this research. Third, ESGD is conceptualized as a mediator which may allow firms to examine the effectiveness of their EMPs and EP contributing to the literature on measuring sustainability and FP. Fourth, the methodological approach of this research is discrete in the context of environmental management studies as the DEA technique is still at a rudimentary phase in environmental studies. Lastly, we use five major proxies (total carbon emission produced, carbon emission intensity, carbon emission productivity, carbon emission per unit size, and DEA) to measure EP and ESG scores of individual components as the proxies of ESGD to analyze its mediating role.

The rest of the study is organized as follows. Section two outlines the theoretical background and hypotheses followed by the research methods in section three. Section four delineates the findings and discussion and finally, section five concludes this study.

## 2 Conceptualizing EMPS and EP

These days organizations face constant pressure from their stakeholders to implement EMPs (Delmas and Toffel, 2008). Generally, different organizations perceive EMPs differently. According to Shrivastava and Hart (1995), EMPs allow organizations to combat perspective environmental issues by acquiring raw materials and implementing environment-friendly practices in packaging and waste disposition. Therefore, EMPs represent a set of organizational practices focused on the conservation of resources by decreasing consumption and improving waste disposal. Organizations also leverage technology in designing products, manufacturing, and managing their waste. Similarly, Monotabon et al. described EMPs as “the set of techniques, policies, and procedures used by the firms to monitor and control the impact of their activities on the natural environment.” EMPs help firms in improving their EP which represents environmental initiatives taken by the firms to streamline their operations in a way that does not harm the environment (Tyteca, 1996; Ulubeyli, 2013). Organizations require dynamic capabilities to implement EMPs (Bowen et al., 2001). Hence, it is crucial for organizations to improve their EP through changes in business processes and by developing unique capabilities. Managers need to focus on developing environment-related capabilities instead of involving in complex environmental initiatives. Earlier studies suggested that managers require proper guidance to develop these capabilities to support EMPs (Bowen et al., 2001) and improve organizational EP.

Earlier studies employed different business perspectives to define EP. According to Lober (1996), EP is an organizational commitment to preserving and protecting their natural environment considering different focus areas including maintenance of water, air, soil, etc. EP has several dimensions which broadly examine the impact on the natural environment, resources, consumption, waste, and emissions resulting due to diverse business activities. Considering EP as a multidimensional concept, Epstein (1996) categorized pollutants and waste reduction, resources and energy conservation, manufacturing safe products, and potential risks as the major dimensions of EP. Another definition of EP was suggested by the Organization for Economic Co-operation and Development (OECD), governments use EP to assess the public sector firms' progress to achieve environmental goals, promote continuous policy dialogue, and peer learning, and stimulate accountability among the firms. The scant literature on EP offers a limited understanding of the concept and has linked societal obligations (Judge and Douglas, 1998), and stakeholders’ expectations (Lankoski, 2000) to EP. Further, researchers employed different approaches to determine the EP of firms ranging from the process, resources consumption, emissions, and waste, efficiency, ecological and financial effects, and the perception of consumers (James, 1994). Another systematic study determined EP by analyzing impact indicators which evaluated the physical and monetary impacts of EP (Bartolomeo, 1995). Recent research developed an exclusive index using 40 performance indicators to measure EP and ranked 180 countries based on their climate change performance, environmental health, and ecosystem vitality (Wolf et al., 2022) Similarly, another study measured EP by designing socioeconomic and environmental indicators (Zhang and Wu, 2022).

### 2.1 Conceptual background

The past studies have applied several conventional theories namely legitimacy, resource dependence, stakeholder, and signaling theories to understand and interpret the linkage between EP and FP (Boakye et al., 2021). The main problem with these conventional theories is the lack of effectiveness to address complex environmental issues and poor recognition of the significance of natural resources as a part of organizations’ social and environmental strategy (Oliver, 1997; Wagner, 2015). Therefore, organizations require a holistic framework to address dynamic environmental issues that can explain different EMPs and link EP and FP. In this regard, institutional and resource-based view (RBV) theories are employed to address dynamic environmental issues faced by the firms and analyze the linkage between EMPs, EP, ESGD, and FP (Tran et al., 2021).

The features of RBV advance that developing internal capabilities helps organizations in gaining a competitive advantage (Barney, 1991). RBV offers an explanation of how firms may develop internal resources. According to Nisar et al. (2021), access to tangible (skilled labour) and intangible (financial and non-financial) resources allows firms to achieve a competitive advantage. In the context of industrial firms, pollution reduction burdens firms’ resources (Porter and Van der Linde, 1995) whereas, these environment-related initiatives achieve sustainability (Al-Tuwaijri et al., 2004; Jung et al., 2018). Hence, RBV requires firms to adopt and develop strategic environmental capabilities to represent good EMPs which will also boost their growth, financial performance, and access to critical resources.

Alternatively, firms' environmental initiatives represent their wider external engagement which can be explained through institutional theory (Berrone et al., 2017). This theory suggests that transformations in social and cultural expectations are highly influenced due to firms’ operational activities (Feng and Wang, 2016). The isomorphism in firms is categorized into coercive, cognitive, and normative types (DiMaggio and Powell, 1983). Institutional isomorphism represents direct (regulatory authorities and governments) and indirect forces (social and cultural expectations) that impact structural and technical components while complying with environmental legitimacy (Liao et al., 2020). A few external forces such as political factors exerted by stakeholders, force firms to undergo environmental scrutiny, fines, closures, and consumers boycotting their products (DiMaggio and Powell, 1983). Normative components argue that social values, institutional affiliations and networks, suppliers, skilled employees, and customers are influenced by the media (Soobaroyen and Ntim, 2013). Consequently, instead of developing environment-specific protocols, firms allocate essential resources to implement EMPs as a part of their wider organizational policy (Wang et al., 2018). This leads to infer that commitment to good EMPs will result in better EP, especially by reducing CO2 emission and pollution. Thus, maintaining good relations with their key stakeholders allow firms to improve their FP.

Based on the complexity of EMPs and the dynamic business environment, it is impossible to rely on a single theoretical framework to explain the economic perspective. Therefore, the current study suggests analyzing the EMPs, EP, ESG, and FP of industrial firms using RBV and institutional theories. This approach responds to the limitations highlighted in the recent studies (Aerts et al., 2008; Feng and Wang, 2016; Moussa et al., 2020) and encouraged the integration of socio-political elements to investigate firms’ environmental and financial prospects.

### 2.2 Relevance of DEA technique

The plethora of literature concentrated on the nexus between the environment and FP of firms has rigorously used DEA which offers an adequate justification to establish the empirical foundation of this study. DEA is useful to evaluate firms' efficiency and use as a diagnostic tool for environmental management decision-making (Emrouznejad et al., 2019; Khodadadipour et al., 2021; Wegner and Amin, 2019). A recent study used two-a stage DEA method to explore the efficiencies of 23 countries through capital, labor, and energy consumption and concluded that Iceland, Luxemburg, and New Zealand were the most efficient countries (Halkos and Argyropoulou, 2021). According to Wei et al. (2021), the current techniques are unable to provide accurate measurement of CO2 emission, therefore DEA combined with stochastic multicriteria acceptability analysis (SMAA-2) are acceptable techniques for the evaluation of energy and environmental efficiencies. DEA technique has also been used in assessing firms’ sustainability of environmental performance (Cui et al., 2021), eco-efficiency and eco productivity (Demiral and Sağlam, 2021; Matsumoto and Chen, 2021), carbon emission performance, and economic determinants (Lv et al., 2021), environmental performance (Albertini et al., 2021; Matsumoto et al., 2020), and haze influencing factors (Zhou et al., 2019). Similarly, DEA coupled with stochastic DEA cross efficiency has been used in the past to design regulatory strategies for the banking industry (Liu et al., 2020; Zhao et al., 2021). Based on the settings of this study and realizing the relevance of DEA to accurately measure the impact of EMPs on EP and FP, it is justified to adopt this method to evaluate the performance of firms, address issues in existing environmental policies, and propose perspective eco-friendly business strategies (Sueyoshi et al., 2017).

### 2.3 Empirical literature and hypotheses

#### 2.3.1 Nexus between EMPs and EP

RBV argues that firms may achieve EP targets by allocating resources and developing capabilities through proper environment-friendly initiatives (Alam et al., 2019). Firms that utilize advanced-level EMPs and structures are able to improve their FP and contribute to environmental protection in two different ways. Firstly, developing capabilities and allocating resources to leverage clean technologies allow firms to harmonize their business according to emerging global markets and facilitate achieving eco-efficiency targets. Secondly, the adoption of relevant EMPs inspires organizations to develop clean and efficient energy policies. Hence, the reduction of CO2 emissions and environmental protection is only possible by embracing and implementing a range of EMPs programs.

Progressive firms have restructured their strategic directions to achieve EP goals by implementing different concepts of EMPs (Ali et al., 2020a; Florida, 1996; Li et al., 2021; Theyel, 2002). The findings of previous studies examining the relationship between EMPs and EP reflect mixed results. The studies on US chemical firms revealed that enhanced EMPs commitment lowers environmental practices resulting in the release of more toxic material in the atmosphere (Delmas and Blass, 2010). Whereas, recent studies contradicted these findings and suggested that firms taking green initiatives may highly improve their green performance (Ali et al., 2020b; Chen et al., 2018; Mungai et al., 2020; Jyoti and Khanna, 2021). This leads to establishing that implementing good EMPs allow firms to minimize the hazardous impact on the ecological system (Du et al., 2019; Wang et al., 2018). Modern-day firms gearing to achieve environmental and social goals need to remain proactive in implementing effective environmental strategies by focusing on reducing carbon emissions (Moussa et al., 2020). Another study on the European hotel industry (Italy, Spain, and Portugal) found that despite obtaining environmental certification European hotels were unable to report significant improvement in EP. While in Malaysia, stakeholders (investors, creditors, government, and environmental agencies) are highly concerned with the contribution of firms toward sustainable development (Atan et al., 2018). This has led the Malaysian government to implement several environmental regulations on industrial firms to protect the environment and enhance the industrial sector's contribution to the sustainable development of the country (Md Nor et al., 2016). Studies on Malaysian firms found that general EMPs under ESG compliance have no significant impact on FP (Atan et al., 2018), however, implementing innovative EMPs and green initiatives have indicated a positive effect on FP (Ong et al., 2019). This argument essentializes investigating the impact of EMPs on EP and examining whether good EMPs result in better EP. Hence, hypothesis one is proposed as follows;

H1 EMPs have a positive effect on the EP of firms.

#### 2.3.2 Nexus between EP and ESGD

Theoretical components of institutional theory indicate that firms may improve their ESGD by committing to good EP as these practices reduce firms’ operational cost, optimize resources and energy consumption, and results in a positive effect on FP. Alternatively, features of RBV argue that firms may achieve competitive advantage and improve their growth by improving their EP allowing them to improve their reputation, brand value, and establish a close relationship with key stakeholders which may positively influence FP (Russo and Fouts, 1997).

Although ESGD is established as a common criterion for firms' ratings, there is a lack of information about firms' responses and the impact of these ratings on their performance (Clementino and Perkins, 2021). The findings of past studies argue that ESGD motivates firms to avoid class action and mitigate financial penalties by improving their EP (Murphy and McGrath, 2013). The literature on ESGD and EP for firms is largely fragmented as a few studies found a significantly positive relationship between EP and ESGD (Alareeni and Hamdan, 2020; Albitar et al., 2020), and others reported a modest relationship between EP and ESGD (Huang, 2021). Besides regulatory compliance, firms tend to remain cautious in the selection of their environmental initiatives and employ different EMPs so that besides regulatory compliance under ESGD, these EMPs improve their sustainability and financial performance (Arun et al., 2022). Over the years, organizations have designed and implemented numerous ESG-based business models to address environmental and financial sustainability issues (Rajesh and Rajendran, 2020). However, the scant literature on ESG offers little information about its integration and strategic implementation at the firm level which may affect the environmental policies of the firms (Lokuwaduge and Heenetigala, 2017). A few scholars have attempted to classify factors of firms' engagement in ESG initiatives and concluded that firms often engage in environmental initiatives to minimize the internal and external stakeholders' pressure by designing strategies for EP which may lead to better ESGD (Russo and Fouts, 1997; Shen et al., 2019). EP motivates firms to continue implementing environment-friendly initiatives by establishing an effective relationship with their stakeholders which reduces their operational costs as well as improves their ESGD (Nguyen et al., 2020). Following RBV's logic, EP can be considered an institutional strategy that may help firms in gaining a competitive advantage by resolving their ESG issues. Consequently, the second hypothesis is proposed as follows;

H2 EP has a positive effect on the ESGD of firms.

#### 2.3.3 Nexus between EP and FP

RBV also suggested that gaining a competitive advantage and improving FP hinge on firms' environmental efficiency fulfilling the sustainability expectations of influential stakeholders, environment conservation, and seeking wider social acceptance (Hart, 1995; Rivera et al., 2017). This indicates that firms' tentative engagement in environmental initiatives is guided by external and internal pressures such as accountability pressure from stakeholders, reputation, and brand value in the global market which often results in positive changes in FP (Russo and Fouts, 1997; Shen et al., 2019). Institutional theory advances a similar narrative of improvement in firms' FP through environmental commitments. Firms’ ongoing commitment to environment-friendly initiatives establishes progressive connections with their stakeholders and significantly reduces operational costs (Nguyen et al., 2020; Zeng et al., 2010).

The findings of past empirical studies analyzing the relationship between EP and FP reported mixed results. Some seminal and a few novel studies claimed that EP and FP are positively associated (Al-Tuwaijri et al., 2004; Ambec and Lanoie, 2008; Konar and Cohen, 2001; Manrique and Martí-Ballester, 2017; Prado-Lorenzo and Garcia-Sanchez, 2010; Russo and Fouts, 1997; Wagner, 2015). These findings were authenticated by recent studies of Rivera et al. (2017) and Aslam et al. (2021) while analyzing the impact of EP on the FP of US-listed and Japanese-listed firms. However, some studies have also claimed that EP and FP of firms have no mutual association and/or negative relationship with each other negative (Cormier and Magnan, 1997; Stanwick and Stanwick, 1998; Earnhart and Lizal, 2007; Qiu et al., 2016; Walls et al., 2012). This indicates that literature on the relationship between EP and FP is scattered and no mutual agreement exists between scholars. The findings of previous studies are inconclusive due to two main factors. First, most of these studies have focused on subjective methods to estimate firms’ EP using disclosure proxies which poorly capture the actual EP of firms (Albertini, 2013; Deegan, 2013, 2017; de Castro Sobrosa Neto et al., 2020). Second, there is well-documented evidence that the effectiveness of environment-friendly initiatives can only be seen in the long term therefore, firms need to stay committed to their environmental activities to gain economic and financial benefits (Busch and Lewandowski, 2017). Whereas, studies analyzing the effect of EP on FP of Malaysian firms lack robustness due to theoretical and sample limitations (Atan et al., 2018; Hazudin et al., 2015; Md Nor et al., 2016; Ong et al., 2019). The government of Malaysia continues to develop various regulations and acts to monitor industrial effluents and their effect on the environment. These regulations are expected to improve the EP of industrial firms which may positively affect FP. Consequently, the third hypothesis predicts that;

H3 EP has a positive effect on FP for firms.

#### 2.3.4 ESGD as the mediator between EP and FP

**Figure 1:**
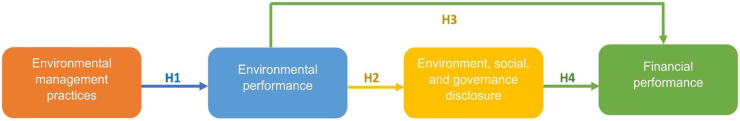
Theoretical model.

The earlier discussion indicates that most of the past studies have focused on analyzing the direct impact of EMPs on the FP of firms and the results of these studies present mixed findings (Florida, 1996; Hertin et al., 2008; Jiang et al., 2018; Jyoti and Khanna, 2021; Miroshnychenko et al., 2017; Montabon et al., 2007; Xie et al., 2019). The findings of these studies are limited due to the failure to incorporate the impact of essential mediators between EMPs, EP, and FP. The institutional theory's theoretical components argue that firms' commitment to strong EMPs increases their opportunities to experience positive changes in EP through investments in environmental initiatives which allow firms to reduce their negative impact on the environment, improve brand value, and result in positive changes in FP (Lin et al., 2013; Porter and Van der Linde, 1995). RBV argues a similar perspective of positive changes in firms' FP by implementing good EMPs which lower production costs, increase productivity and firms efficiently utilize their resources (Bernauer et al., 2007). RBV also suggests that commitment towards good EMPs represents firms' broad commitment toward corporate social responsibility (CSR) policy which should be disclosed in corporates' nonfinancial reporting (Raimo et al., 2020) hence, corporates are required to establish certain standards and indicators to estimate EMPs and EP (Kassem et al., 2017). As suggested by institutional theory, commitment toward good EMPs improves EP by investing in environmental initiatives and allocation of resources. However, powerful stakeholders often require firms to disclose their environmental, social, and governance (ESG) information so that investment decisions are made accordingly (Chouaibi et al., 2021). ESG disclosure (ESGD) incorporates ratings of the firms based on their EP therefore, firms concerned with the reputation of their brand value concentrate on improving their ratings. Past studies have associated ESGD with firms' operating performance and operational costs (Ruan and Liu, 2021). Additionally, it is also confirmed that ESGD reduces firms' risk-taking behavior and lowers their financing cost (Di Tommaso and Thornton, 2020; Oikonomou et al., 2014) which will avoid spending on unnecessary EMPs. This leads us to infer that firms with better ESGD ratings represent better EP which results in better FP therefore, it is predicted that the relationship between EP and FP is essentially mediated by ESGD (Figure 1) which leads us to propose the fourth hypothesis as follows;

H4 ESGD mediates the relationship between EP and FP of the firms.

## 3 Materials and methods

### 3.1 Data and sampling

This study covers the FP of Malaysian firms between 2009–2020. The selection of the study period is important to estimate the constructs of this study as the Ministry of Environment and Water promulgated ‘Industrial Effluent Regulations 2009’ which required Malaysian industrial firms no implement best management practices to prevent and reduce the discharge of industrial effluent contaminating the environment. Hence, the study period is projected to estimate the actual effect of EMPs, EP, and ESGD on FP. Initially, the sample contained 1764 firm-year observations of 147 firms from 13 different industries listed on Bursa Malaysia. However, in the final sample, 6 firms from different sectors (real estate and finance) were excluded for comparison of results also, these firms had mandated regulatory and reporting patterns (Ntim, 2016). Additionally, excluded firms were not significant contributors to environmental pollution and energy consumption. During data screening, we excluded 227 firm-year observations due to a lack of information about carbon emissions and EMPs during the sample selection period. The final sample contained an unbalanced panel dataset of 1537 firm-year observations of 141 firms in 10 different industries between 2009–2020. The descriptive statistics of firms with industry types are reported in Table 1. We used three popular databases i.e., Thomson Reuters Assets 4, Worldscope, and Bloomberg to extract data related to EMPs, EP, FP, and ESGD as these databases are authentic data sources in the world press. Table 1 shows that the industrial products & services industry had 405 (26.35%) firm-year observations, followed by the energy 246 (16%), and transportation and logistics 172 (11.11%) industries.

**Table 1:** Classifying the firms into relative industries.

Industries	f	%
Industrial products & Services	405	26.35
Energy	246	16
Transportation & Logistics	172	11.11
Utilities	134	8.71
Technology	125	8.13
Health care	119	7.74
Consumer products & Services	106	6.89
Construction	102	6.63
Telecommunication & Media	73	4.74
Plantation	55	3.57
Total	1537	100

### 3.2 Experimental variables

To test the hypotheses of this study, we operationalized four main variables. EMPs is an explanatory variable estimated using a scale developed by Xie and Hayase (2007), Trumpp et al. (2015), and Aslam et al. (2021). This scale is comprised of five sub-dimensions of EMPs with 29 items covering broad areas of environmental policy (8 items), environmental objectives (5 items), environmental processes (6 items), organizational structure (4 items), and environmental monitoring (6 items). The items of the scale were imported from Xie and Hayase (2007), Trumpp et al. (2015), and Aslam et al. (2021) and were modified to suit the context of this study. We used this measurement scale to estimate the EMPs index by adding scores of dimensions items (1 represents an environmental initiative taken by the firm, 0 no initiative taken). Similarly, EP scores are estimated by following the same criteria to represent the level of EP quality which ranges from 29 (implementing good EMPs) to 0 (implementing poor EMPs). The developed scale elucidates all the aspects of firms’ environmental initiatives promulgated by ISO 14001. The scale operationalized to measure EMPs is presented in Table 2.

**Table 2:** Operationalizing EMPs scale.

Sub-dimensions	Items	Source
Environmental policy	1. Firm has the policy to improve efficiency, resources, and packaging.	Xie and Hayase (2007)
	2. Firm has established a policy for inclusive efficiency of resources.	
	3. Firm has a policy to improve sustainable packaging.	
	4. Firm has a policy to improve its water efficiency.	
	5. Firm has a policy to minimize the environmental impact of its supply chains.	
	6. Firm has a dematerializing policy.	
	7. Firm has an eco-friendly design policy.	
	8. Firm has a policy to assess a product's life cycle.	
Environmental objectives	1. Firm has well-established targets of energy efficiency.	Aslam et al. (2021)
	2. Firm has well-established targets of general resources' efficiency.	
	3. Firm has well-established targets to use sustainable packaging.	
	4. Firm has well-established targets of water efficiency.	
	5. Firm has well-established targets of supply chain impact on the environment.	
Environmental Processes	1. Firm uses environmental selection criteria such as ISO 14000, energy consumption, etc to select its suppliers and sourcing partners.	Trumpp et al. (2015)
	2. Firm describes and claims to have an established supply chain as the firm's effort to reduce overall environmental impact.	
	3. Firm claims to utilize environmental criteria (lifecycle assessment) for sourcing and eliminating materials.	
	4. Firm describes and claims to have established processes for improving energy efficiency.	
	5. Firm describes and claims to have established processes for improving resources efficiency.	
	6. Firm describes and claims to have established processes for improving sustainable packaging.	
Organizational structure	1. Firm offers environmental issues training to its employees.	Trumpp et al. (2015)
	2. Firm has a designated team for environmental management.	
	3. Firm claims to have an EMS certification.	
	4. Firm describes and claims to have established processes for maintaining environmental management.	
Environmental monitoring	1. Firm claims to use key performance indicators (KPI) or balanced scorecards to assess energy efficiency.	Trumpp et al. (2015)
	2. Firm claims to use KPI or balance scorecard to assess resource efficiency.	
	3. Firm claims to use KPI or balance scorecard to assess sustainable packaging usage.	
	4. Firm claims to use KPI or balance scorecard to assess water efficiency.	
	5. Firm claims to use KPI or balance scorecard to assess the supply chain's environmental impact.	
	6. Firm conducts surveys of its suppliers' environmental performance.	

Note: score 1 is given if the information is available; 0, if no information is available.

EP is a response variable estimated by five unique proxies. These proxies are environmental efficiency (EP_DEA) representing total energy consumption (input) and sales (good output) and total carbon emission (bad output) (Chen et al., 2017; Song et al., 2018). According to (Chen et al. 2017), environmental efficiency can be measured through nonparametric techniques such as DEA, which considers a range of inputs into several different outputs (desired and undesired) and exclude previously established assumptions about the relationships between inputs and outputs. Following these criteria, firms' environmental efficiency is estimated by applying a constant return to scale input DEA efficiency. Based on the sensitivity to the environment, industries in the initial sample were divided into two types (sensitive and non-sensitive) which helped in estimating firms' efficiencies each year. Carbon emission productivity (EP_EE) was the second proxy of EP which was estimated by net sales to total carbon emission (Chen et al., 2018). Whereas, carbon emission per unit size of total assets (EP_PUS) and carbon emission produced (EP_Emi) were the third and fourth proxies of EP which were developed using Mungai et al. (2020) and Moussa et al. (2020) criteria. Finally, the intensity of carbon emission (EP_Int) was the fifth proxy of EP which was measured using carbon emission/sales following Trumpp and Guenther's (2017) criteria. We separated these proxies based on their expected association with EMPs, FP, and ESGD.

FP is also a response variable estimated by Tobin's Q technique. The past studies analyzing the effects of EMPs on firms' FP have vigorously used this technique (Busch and Lewandowski, 2017; Horváthová, 2010; Manrique and Martí-Ballester, 2017; Shen et al., 2019; Surroca et al., 2010; de Castro Sobrosa Neto et al., 2020) and concluded that Tobin Q is a reliable technique to estimate the actual effect of firms' long-term investments (related to environment-friendly initiatives) (Dowell et al., 2000; Surroca et al., 2010) and it is less sensitive to management's manipulation as compared to other accounting methods (Hassan and Romilly, 2018).

ESGD is a mediator between EP and FP of firms which were estimated by a proxy of ESG ratings developed by Bloomberg. This method is credible and commonly used in business studies and follows Conca et al. (2021) criteria. The main reason to prefer Bloomberg's indicators is due to the estimation method which directly calculates ESG based on relative information disclosure instead of relying on empirical models. ESGD scores were split into single components (E, S, and G) which allowed us to estimate whether disclosure of individual E, S, and G components (environment, social, and governance) mediate the relationship between EP and FP (1, represents ESG's individual component is disclosed; 0, no disclosure available).

Finally, firm-specific characteristics such as size, clean technology, leverage, research and development (R&D), and industry sensitiveness to pollution are used as control variables adopted from extant literature on environmental studies (Liao, 2018; Liao et al., 2020; Hassan and Romilly, 2018; Moussa et al., 2020). The size of firms is estimated by the natural log of firms' total assets (Haque and Ntim, 2020; Moussa et al., 2020). Past studies suggested that implementing environment-friendly initiatives in large firms is guided by their financial freedom to use clean energy for the reduction of carbon emissions (Moussa et al., 2020; Onubi et al., 2020; Jung et al., 2018). Similarly, implementing environment-friendly initiatives in small firms is guided by their flexible and non-hierarchical organizational structure (Halkos and Tzeremes (2007). Past studies have confirmed that cleaner and green technologies allow firms to reduce carbon emissions which positively influence firms' EP (Blackman and Bannister, 1998). The data related to clean technology usage (1, one use of clean technologies; 0, no use) was retrieved from the Thomson Reuters Assset4 database. In the context of leverage, past studies revealed that firms with high leverage enhance their commitment to good EMPs which allows them to meet stakeholders' expectations and have a positive impact on EP and FP (Moussa et al., 2020). Firms' leverage is measured by total debt divided by total assets following Shahab et al.'s (2020) criteria. Alternatively, investment in R&D was found to have a positive impact on firms’ EP and FP as it improves resource management and reduces carbon emissions (Alam et al., 2019). We used a natural log of total R&D expenditure to estimate R&D. Lastly, Moussa et al. (2020) predicted that industry type can be linked to EMPs and EP, as environment-sensitive firms are likely to disclose more information regarding environment and social performance as compared to non-sensitive firms (Qureshi et al., 2020). Industrial context is estimated by following Qureshi et al. (2020) criteria (1 represents environment sensitivity and 0 non-sensitivity). All these variables with respective estimations are reported in Table 3.

**Table 3:** Variables’ estimations.

Variables	Type	Symbol	Effect	Source	Measurements
Environmental management practices	Independent	EMPs	±	Assets 4	EMPs was calculated by adding 29 items presented in Table 2. EMPs score ranges from 0 to 29.
Environmental performance	Dependent	EP	±	Assets 4	EP was measured by five different proxies as explained under variables and measurements.
Financial performance	Dependent	FP	±	Worldscope	FP was estimated by market value of total shares + total liabilities divided by total assets
Environment, social, governance disclosure	Mediating	ESGD		Bloomberg	ESGD was estimated by considering overall score of ESGD and then these scores were split (E, S, and G) to analyze the mediating effect of each issue between EP and FP.
Size	Control	F_Size	±	Worldscope	Natural log of firm's total assets.
Clean technology	Control	C_Tech	±	Assets 4	1, C_Tech is used; 0, C_Tech not used.
Leverage	Control	Lev	±	Worldscope	Total debt divided by total assets.
Research and Development	Control	R&D	±	Worldscope	Log. of total R&D expenditure.
Industry sensitiveness	Control	Polluters	±		1, firm is sensitive to environment; 0, firm is not sensitive to environment.

### 3.3 Estimation models

While measuring the linkage between EMPs, EP, ESGD, and FP, it is essential to resolve endogeneity issues so that the findings are reliable and valid. According to (Ullah et al. 2018), endogeneity issues during statistical analysis can be resolved by a generalized method of moments (GMM). This technique uses internal instruments by deriving lagged values of dependent variables (Blundell and Bond, 1998). Additionally, the data loss during estimation can be recovered by a two-step GMM model (Ullah et al., 2018). Therefore, this study corroborated the dynamic two-step GMM model to resolve endogeneity and causality issues during model estimation which is consistent with the recent studies examining EMPs, EP, and FP (Aslam et al., 2021; Haque and Ntim, 2018).

To examine the effect of EMPs on EP (H1), the GMM regression estimation model is as follows;

EPit=β0+β1EPit−1+β2EMPsit+β3Cit+μit+εit

While, EP represents environmental performance estimated using five proxies (EP_DEA; EP_EE; EP_PUS; EP_Emi; EP_Int) and as alternative response variables; β is the coefficient of the degree of change; β1EPit – 1 represents first legged of the dependent variable; EMPs is the explanatory variable which signifies environmental management practices, C indicates control variables; i represents each firm, t is year, μ is the time effect (fixed), and git is the error term.

The effect of EP on the ESGD of firms (H2) is analyzed through the following regression model;

ESGDit=β0+β1ESGDit−1+β2EPsit+β3Cit+μit+εit

While, ESGD refers to the environment, social, and governance disclosure; ESGDit – 1 is first lagged of the dependent variable; EP determines environmental performance; C signifies control variables as mentioned above.

The effect of EP on FP (H3) is analyzed using the following regression model;

FPit=β0+β1FPit−1+β2EPit+β3Cit+μit+εit

While, FP indicates the financial performance of firms; FPit – 1 represents first lagged of outcome variable; EP determines environmental performance; C represents control variables.

The mediating effect of ESGD between EP and FP (H4) is estimated using the following regression model;

FPit=β0+β1FPit−1+β2ESGDit+β3EPit+β4Cit+μit+εit

While, FP denotes firms’ financial performance; FPit – 1 represents first lagged of the dependent variable; ESGD is the mediator which represents the environment, social, and governance disclosure; C represents firm-specific characteristics (control variables).

## 4 Results

### 4.1 Descriptive statistics and correlation analysis

Table 4 outlines the descriptive statistics and univariate analysis of the variables. The mean value of the main (explanatory) variable is 12.23 with a standard deviation of 5.15 which confirmed that most Malaysian firms have adopted environmental management practices to protect the environment and reduce carbon emissions. These findings are consistent with the past studies of Atan et al. (2018) and Ong et al. (2019) revealed that Malaysian industrial firms tend to implement innovative EMPs. While, mean values of dependent variables FP and EP including all five proxies of EP (EP_DEA, EP_EE, EE_Emi, EP_Int, and EP_PUS) are widespread (less clustered). In contrast, the mean and standard deviation values of ESGD and its three proxies (E, S, and G) are less spread and appear more clustered around the mean.

**Table 4:** Descriptive statistics of observed variables.

Variable	Observations	Mean	Std.Dev	Minimum	Maximum
*Dependent*					
FP	1501	2.16	1.10	1.13	9.87
EP_DEA	1495	0.74	0.24	0.56	1.00
EP_EE	1487	3.48	1.62	3.25	9.21
EP_PUS	1492	−0.23	0.05	−0.82	1.23
EP_Emi	1504	10.49	2.23	4.22	12.92
EP_Int	1482	1.46	0.37	1.45	6.67
EP	1421	2.86	1.16	1.89	10.45
*Independent*					
EMPs	1537	12.23	5.15	0.00	29.00
*Mediating*					
ESGD	1493	0.45	0.14	0.38	1.00
E_D	1493	0.58	0.36	0.27	0.95
S_D	1493	1.34	0.54	0.46	1.98
G_D	1493	0.90	0.48	0.26	1.89
*Control*					
F_Size	1496	20.14	1.12	17.78	23.54
C_Tech	1496	1.40	0.76	0.00	1.00
Lev	1496	2.76	1.23	0.55	2.85
R&D	1496	16.43	3.36	9.58	23.20
Polluters	1496	1.83	1.14	0.00	1.00

The results of the correlation between variables are reported in Table 5. The correlation between EMPs and EP is significantly positive confirming that the results comply with earlier assumptions (implementing effective EMPs has a positive effect on EP) (H1). Similarly, it is notable from the correlation matrix that EP and ESGD have a significant positive correlation. This result complies with our prediction in H2 (EP has a positive effect on ESGD). The correlation between EP and FP is significant and positive confirming the compliance of this result with our prediction in H3. In terms of ESGD and its individual components, the correlation results are significant and positive except for governance proxy (G_D). Overall, the correlation analysis determines that the results broadly comply with assumptions established under hypotheses (H1, H2, H3, and H4). Finally, the correlation between independent (EMPs) and control variables (F_size, C_Tech, Lev, R&D, and polluters) is low which indicates that our models are less likely to suffer from multicollinearity issues.

**Table 5:** Correlation analysis.

	FP	EP_DEA	EP_EE	EP_PUS	EP_Emi	EP_Int	EP	EMPs	ESGD	E_D	S_D	G_D	F_Size	C_Tech	Lev	R&D	Polluters
FP	1.00																
EP_DEA	0.07∗	1.00															
EP_EE	0.15∗	0.45∗	1.00														
EP_PUS	-0.10	-0.17	0.14∗	1.00													
EP_Emi	0.23∗	0.47∗	0.26∗	-0.07	1.00												
EP_Int	-0.14∗	-0.05∗	-0.43∗	-0.40	0.70∗	1.00											
EP	0.36∗	0.41∗	0.47∗	0.34∗	0.30	0.28∗	1.00										
EMPs	0.10∗	0.36∗	-0.28∗	0.12∗	-0.08∗	0.44∗	0.31∗	1.00									
ESGD	0.10∗	0.13∗	0.42∗	-0.09∗	0.27	0.22	-0.25	0.32∗	1.00								
E_D	0.09	0.10∗	0.17∗	0.24∗	-0.16	0.31∗	-0.52	0.38	0.65∗	1.00							
S_D	0.16∗	-0.10∗	0.14∗	-0.23∗	0.42	0.30∗	0.20∗	0.38∗	0.27∗	0.41∗	1.00						
G_D	0.06	0.08	0.08∗	0.13∗	0.23∗	0.26	0.22	0.38	0.34	0.63	0.56∗	1.00					
F_Size	0.14∗	0.18∗	-0.26∗	0.43∗	0.56	0.88∗	0.32	-0.09∗	-0.17∗	0.62∗	0.73	0.54∗	1.00				
C_Tech	0.08∗	0.52∗	0.27∗	0.32	0.19	0.35	0.48	0.42∗	0.46∗	0.54∗	0.42∗	0.38∗	0.61∗	1.00			
Lev	0.17	-0.09∗	0.32∗	0.19∗	0.14	0.16∗	0.23∗	0.26∗	0.12∗	0.24∗	0.37∗	0.38∗	0.38∗	0.28∗	1.00		
R&D	0.32∗	-0.27∗	-0.29	-0.37	-0.32	-0.35	-0.39	0.13∗	0.15∗	0.18∗	0.11∗	0.47	0.42	0.39∗	0.40∗	1.00	
Polluters	-0.07	-0.12	-0.15	-0.18	-0.47	-0.34	0.16∗	-0.25∗	0.27∗	-0.42∗	0.13∗	-0.33∗	-0.61	-0.66	-0.47	-0.41	1.00

∗ Represents statistical significance at a 5% level.

### 4.2 Hypothesis testing

The hypotheses were tested through a two-step GMM regression analysis by controlling endogeneities of dynamic, simultaneous, and omitted variables (Erdogdu, 2011; Ullah et al., 2018). First, we detected serial autocorrelation (AR) issues in the models using *p* values. Generally, *p* values of AR confirm whether the models have serial autocorrelation problems. The results of the two-step GMM regression are reported in Table 6. The *p* values of AR (1) are significant whereas, AR (2) are insignificant which corroborates that our models do not contain serious serial autocorrelation issues. After addressing the endogeneity issues, we performed GMM regression analysis to analyze the effects of EMPs on EP (H1), EP on ESGD (H2), and EP on FP (H3). The results of Two-step GMM estimations (Table 6) delineate that EMPs were measured using 29 indicators, EP through five proxies (EP_DEA, EP_EE, EP_PUS, EP_Emi, and EP_Int), and ESGD through three proxies (E_D, S_D, and G_D). Three proxies of EP (EP_DEA, EP_EE, and EP_PUS) predicted a positive association with EMPs, ESGD, and FP while the other two proxies (EP_Emi and EP_Int) predicted negative interaction with EMPs, ESGD, and FP (Table 6). Altogether, 16 models were tested to examine the relationship between EMPs and EP, EP and ESGD, and ESGD with EP and FP.

**Table 6:** Results of GMM regression analysis.

Dependent variables										
Variables	Model 1 EP_DEA	Model 2 FP	Model 3 FP	Model 4 FP	Model 5 EP_EE	Model 6 FP	Model 7 FP	Model 8 EP_PUS	Model 9 FP	Model 10 FP
Lagged of dependent variables	0.054∗∗∗ (0.002)	0.047∗∗∗ (0.004)	0.475∗∗∗ (0.008)	0.562∗∗∗ (0.010)	0.756∗∗∗ (0.002)	0.457∗∗∗ (0.006)	0.486∗∗∗ (0.004)	-0.047∗∗∗ (0.007)	0.454∗∗∗ (0.010)	0.481∗∗∗ (0.012)
+EMPs	0.004∗∗∗ (0.0007)	0.008∗∗∗ (0.002)		0.005∗∗∗ (0.001)	0.007∗∗∗ (0.002)		0.010∗∗∗ (0.001)	0.009∗∗∗ (0.001)		0.023∗∗∗ (0.000)
E_D	0.048∗∗∗ (0.002)	0.039∗∗∗ (0.001)								
S_D					0.065∗∗∗ (0.004)	0.018∗∗∗ (0.001)				
G_D								0.044∗∗∗ (0.002)	0.031∗∗ (0.001)	
ESGD									0.028∗∗∗ (0.001)	0.051∗∗∗ (0.003)
EP_DEA			0.020∗∗∗ (0.003)	0.226∗∗∗ (0.009)						
EP_EE						0.068∗∗∗ (0.004)	0.063∗∗∗ (0.002)			
EP_PUS									-0.287∗∗∗ (0.008)	-0.264∗∗∗ (0.002)
F_Size	0.027∗∗∗ (0.006)	-0.047∗∗∗ (0.008)	-0.063∗∗∗ (0.001)	-0.042∗∗∗ (0.006)	0.053∗∗∗ (0.010)	-0.073∗∗∗ (0.004)	-0.048∗∗∗ (0.010)	0.069∗∗∗ (0.009)	-0.0342∗∗∗ (0.004)	-0.017∗∗∗ (0.002)
C_Tech	0.033∗∗∗ (0.008)	0.027∗∗∗ (0.003)	0.036∗∗∗ (0.010)	0.047∗∗∗ (0.004)	-0.024∗∗∗ (0.005)	0.063∗∗∗ (0.002)	0.010∗∗∗ (0.000)	0.018∗∗∗ (0.001)	0.042∗∗∗ (0.012)	0.034∗∗∗ (0.006)
Lev	0.066∗∗∗ (0.010)	0.058∗∗∗ (0.004)	0.072∗∗∗ (0.003)	0.056∗∗∗ (0.013)	-0.683∗∗∗ (0.025)	0.0832∗∗∗ (0.015)	0.094∗∗∗ (0.007)	0.063∗∗∗ (0.009)	0.078∗∗∗ (0.000)	0.065∗∗∗ (0.003)
R&D	0.028∗∗ (0.010)	0.024∗∗∗ (0.007)	0.018∗∗∗ (0.003)	0.047∗∗∗ (0.014)	0.053∗∗∗ (0.014)	0.043∗∗∗ (0.018)	0.049∗∗∗ (0.008)	0.071∗∗∗ (0.034)	0.026∗∗∗ (0.005)	0.022∗∗∗ (0.002)
Polluters	-0.021∗∗∗ (0.006)	0.018∗∗∗ (0.003)	0.024∗∗∗ (0.010)	0.028∗∗∗ (0.003)	0.032∗∗∗ (0.015)	0.031∗∗∗ (0.009)	0.035∗∗∗ (0.014)	0.009∗∗ (0.012)	0.025∗∗∗ (0.006)	0.016∗∗∗ (0.000)
Constant	-0.273∗∗∗ (0.037)	0.824∗∗∗ (0.071)	0.927∗∗∗ (0.062)	0.735∗∗∗ (0.062)	0.687∗∗∗ (0.043)	-0.469∗∗∗ (0.024)	0.786∗∗∗ (0.043)	0.729∗∗∗ (0.053)	0.507∗∗∗ (0.038)	0.494∗∗∗ (0.025)
Yearly effect	Yes	Yes	Yes	Yes	Yes	Yes	Yes	Yes	Yes	Yes
AR (1) *p* values	-2.14 (0.03)	-2.52 (0.02)	-2.17 (0.01)	-2.26 (0.03)	-2.86 (0.01)	-2.51 (0.04)	-2.28 (0.00)	-5.78 (0.04)	-2.31 (0.02)	-2.39 (0.01)
AR (2) *p* values	-2.51 (0.14)	0.43 (0.48)	0.52 (0.40)	0.72 (0.58)	0.57 (0.62)	0.41 (0.67)	0.66 (0.74)	-0.87 (0.36)	0.24 (0.62)	0.37 (0.50)
Hansen test's p values	0.45	0.53	0.58	0.55	0.56	0.42	0.47	0.50	0.49	0.52
Obs.	1495	1501	1501	1501	1487	1501	1501	1492	1501	1501
Total firms	141	141	141	141	141	141	141	141	141	141

Dependent variables						
Variables	Model 11 EP_Emi	Model 12 FP	Model 13 FP	Model 14 EP_Int	Model 15 FP	Model 16 FP
Lagged of dependent variables	0.847∗∗∗ (0.003)	0.476∗∗∗ (0.008)	0.484∗∗∗ (0.005)	0.796∗∗∗ (0.006)	0.464∗∗∗ (0.010)	0.456∗∗∗ (0.002)
EMPs	-0.010∗∗∗ (0.001)		0.006∗∗∗ (0.001)	-0.002∗∗∗ (0.001)		0.003∗ (0.001)
E_D	-0.023∗∗ (0.002)	0.010∗∗∗ (0.001)				
S_D				-0.016∗∗∗ (0.003)	0.008∗∗∗ (0.001)	
G_D				-0.019∗∗∗ (0.001)	0.682∗∗∗ (0.009)	
ESGD					-0.012∗∗∗ (0.004)	0.412∗∗∗ (0.004)
EP_Emi		-0.045∗∗∗ (0.004)	0.038∗∗∗ (0.003)			
EP_Int					-0.258∗∗∗ (0.002)	-0.246∗∗∗ (0.008)
F_Size	0.067∗∗∗ (0.006)	-0.001∗∗∗ (0.005)	-0.023∗∗∗ (0.007)	0.053∗∗∗ (0.004)	-0.026∗∗∗ (0.003)	0.063∗∗∗ (0.012)
C_Tech	-0.038∗∗∗ (0.006)	0.093∗∗∗ (0.007)	0.084∗∗∗ (0.009)	0.048∗∗∗ (0.001)	0.075∗∗∗ (0.008)	0.010∗∗∗ (0.000)
Lev	0.237∗∗∗ (0.045)	0.912∗∗∗ (0.026)	0.830∗∗∗ (0.030)	0.434∗∗∗ (0.009)	0.822∗∗∗ (0.021)	0.987∗∗∗ (0.035)
R&D	-0.004 (0.004)	0.069∗∗∗ (0.001)	0.057∗∗∗ (0.003)	-0.026∗∗∗ (0.008)	0.056∗∗∗ (0.002)	0.041∗∗∗ (0.007)
Polluters	0.218∗∗∗ (0.010)	0.009∗∗∗ (0.015)	0.042∗∗∗ (0.028)	0.057∗∗∗ (0.004)	0.048∗∗∗ (0.025)	0.078∗∗∗ (0.025)
Constant	-0.358∗∗∗ (0.221)	0.098 (0.223)	0.524∗∗∗ (0.261)	-0.795∗∗∗ (0.041)	0.312∗∗∗ (0.213)	0.634∗∗∗ (0.269)
Yearly effect	Yes	Yes	Yes	Yes	Yes	Yes
AR (1) p values	-3.28 (0.01)	-3.53 (0.01)	-2.56 (0.01)	-2.83 (0.00)	-2.50 (0.01)	-3.55 (0.01)
AR (2) p values	-2.32 (0.34)	0.40 (0.82)	0.58 (0.74)	2.24 (0.38)	0.43 (0.86)	0.49 (0.82)
Hansen test's p values	0.68	0.57	0.96	0.47	0.53	0.92
Obs.	1504	1501	1501	1482	1501	1501
Total firms	141	141	141	141	141	141

Note. The definitions and explanations of variables are available in Table 3.

∗ Represents statistical significance at 5% level.

∗∗ Represents statistical significance at 10% level.

∗∗∗ Represents statistical significance at 1% level.

The mediating role of ESGD is analyzed by referring to H4 which predicts that ESGD mediates the relationship between EP and FP and the relationship is stronger in firms with better ESGD. The mediator effect is examined by following the hierarchical technique developed by Baron and Kenny (1986). This criterion is fulfilled provided independent variables (EMPs) have a significant impact on dependent variables (EP and FP), independent variables (EP) have a significant impact on mediating variable (ESGD), and finally mediating variables (ESGD) have a significant impact on dependent variables (FP). After fulfilling these conditions, the combined effect of EMPs and ESGD on EP and FP was examined.

The results highlight that mediation conditions are satisfactory (Table 6). The combined regression results of the impact of independent (EMPs) and mediating variable (ESGD) on dependent variables (EP and FP) are reported in Table 6 (models 4, 7, 10 13, and 16). The coefficients related to the effect of EMPs on EP and FP (β = 0.005, p < 0.05; β = 0.010, p < 0.05; β = 0.023, p < 0.01; β = 0.006, p < 0.01; β = 0.003 p < 0.10) are significant and varies (positive, negative) based on the proxy used for EP measurement. Further, it was noticeable that coefficients related to the effect of EP on ESGD (model 2, 6, 9, 12, and 15) were significant (β = 0.039, p < 0.01; β = 0.018, p < 0.05; β = 0.031, p < 0.01; β = 0.010, p < 0.01; β = 0.012 p < 0.01) and fluctuated (positive/negative) based on the effect of relative EP proxy. While, ESGD as a mediator between EP and FP statistically remains significant and varies (positive, negative) based on the disclosure score of relative proxy (E_D, S_D, G_D). To examine the mediating effect of ESGD between EP and FP, we performed the Sobel test following Baron and Kenny's (1986) technique, and the results of the mediation effect together with path coefficients are reported in Table 7. Overall, the mediation effect of ESGD and its three proxies appears significant and positive except for EP∗S_D∗FP.

**Table 7:** Results of mediating effect of ESGD.

Path descriptions	Coefficients	Standard estimates	t-statistics
EP → ESGD → FP	0.0023∗∗∗	0.0002	2.5212
EP → E_D → FP	0.0057∗∗	0.0004	5.8587
EP → S_D → FP	-0.0019∗∗∗	0.0001	-3.5724
EP → G_D → FP	0.0039∗∗∗	0.0002	4.7647

Note. The definitions of variables are presented in Table 3.

∗∗Represents significance at a 5% level.

∗∗∗Represents significance at 1% level.

### 4.3 Robustness checks

To examine the robustness of the results, we performed two robustness checks. The first robustness check involved the measurement of certain EMPs frequently employed by the firms. This was estimated by reviewing the values of the items in the index representing frequently taken environmental initiatives of the firms. The items for environmental policy (EPL) represent the highest values in the index indicating that firms strictly comply with environmental regulations by designing relative EPL. We used similar criteria to estimate EPL by adding the values of the items and constructed an index to measure its impact on EP, ESGD, and FP. The results in Table 8 disseminate that EPL has a significant (positive/negative) effect on EP (models 1, 5, 8, 11, and 14). Further, coefficients for models 1, 5, 8, 11, and 14 are also significant (positive/negative) indicating EP affects ESGD. Similarly, the results (models 3 and 6) show EP's positive effect on FP confirming that our findings are consistent with the results reported in Table 6.

**Table 8:** Results of GMM regression (robustness checks – alternate variable 1).

Dependent variables										
Variables	Model 1 EP_DEA	Model 2 FP	Model 3 FP	Model 4 FP	Model 5 EP_EE	Model 6 FP	Model 7 FP	Model 8 EP_PUS	Model 9 FP	Model 10 FP
Lagged of dependent variables	0.043∗∗∗ (0.003)	0.046∗∗∗ (0.002)	0.403∗∗∗ (0.006)	0.455∗∗∗ (0.010)	0.562∗∗∗ (0.005)	0.541∗∗∗ (0.001)	0.537∗∗∗ (0.003)	-0.056∗∗∗ (0.007)	0.505∗∗∗ (0.006)	0.528∗∗∗ (0.010)
EPL	0.008∗∗∗ (0.001)	0.016∗∗∗ (0.007)		0.004∗∗∗ (0.002)	0.010∗∗∗ (0.004)		0.014∗∗∗ (0.003)	0.011∗∗∗ (0.004)		0.019∗∗∗ (0.004)
E_D	0.048∗∗∗ (0.002)	0.039∗∗∗ (0.001)								
S_D					0.048∗∗∗ (0.010)	0.026∗∗∗ (0.003)				
G_D								0.033∗∗∗ (0.005)	0.036∗∗ (0.002)	
ESGD									0.042∗∗∗ (0.005)	0.046∗∗∗ (0.006)
EP_DEA			0.031∗∗∗ (0.011)	0.038∗∗∗ (0.001)						
EP_EE						0.062∗∗∗ (0.005)	0.066∗∗∗ (0.009)			
EP_PUS									-0.424∗∗∗ (0.006)	-0.414∗∗∗ (0.001)
F_Size	0.019∗∗∗ (0.002)	-0.038∗∗∗ (0.002)	-0.044∗∗∗ (0.003)	-0.048∗∗∗ (0.004)	0.029∗∗∗ (0.006)	-0.030∗∗∗ (0.012)	-0.031∗∗∗ (0.006)	0.040∗∗∗ (0.004)	-0.031∗∗∗ (0.008)	-0.036∗∗∗ (0.003)
C_Tech	0.052∗∗∗ (0.004)	0.047∗∗∗ (0.002)	0.048∗∗∗ (0.006)	0.043∗∗∗ (0.001)	-0.036∗∗∗ (0.006)	0.040∗∗∗ (0.007)	0.039∗∗∗ (0.006)	0.028∗∗∗ (0.010)	0.037∗∗∗ (0.011)	0.033∗∗∗ (0.004)
Lev	0.052∗∗∗ (0.006)	0.055∗∗∗ (0.002)	0.053∗∗∗ (0.003)	0.046∗∗∗ (0.006)	-0.404∗∗∗ (0.011)	0.032∗∗∗ (0.003)	0.041∗∗∗ (0.001)	0.042∗∗∗ (0.006)	0.028∗∗∗ (0.008)	0.045∗∗∗ (0.002)
R&D	0.018∗∗ (0.001)	0.015∗∗∗ (0.002)	0.017∗∗∗ (0.001)	0.014∗∗∗ (0.003)	0.015∗∗∗ (0.002)	0.016∗∗∗ (0.003)	0.012∗∗∗ (0.001)	0.011∗∗∗ (0.003)	0.021∗∗∗ (0.003)	0.018∗∗∗ (0.001)
Polluters	-0.031∗∗∗ (0.004)	0.037∗∗∗ (0.002)	0.035∗∗∗ (0.005)	0.030∗∗∗ (0.001)	0.032∗∗∗ (0.003)	0.034∗∗∗ (0.002)	0.033∗∗∗ (0.003)	0.038∗∗ (0.010)	0.034∗∗∗ (0.003)	0.026∗∗∗ (0.007)
Constant	-0.362∗∗∗ (0.026)	0.713∗∗∗ (0.069)	0.816∗∗∗ (0.051)	0.624∗∗∗ (0.051)	0.576∗∗∗ (0.032)	-0.358∗∗∗ (0.013)	0.675∗∗∗ (0.032)	0.618∗∗∗ (0.042)	0.496∗∗∗ (0.027)	0.383∗∗∗ (0.014)
Yearly effect	Yes	Yes	Yes	Yes	Yes	Yes	Yes	Yes	Yes	Yes
AR (1) *p* values	-2.16 (0.01)	-2.18 (0.01)	-2.22 (0.02)	-2.18 (0.04)	-2.16 (0.01)	-2.31 (0.02)	-2.27 (0.03)	-4.12 (0.03)	-2.43 (0.01)	-2.36 (0.02)
AR (2) *p* values	-2.32 (0.11)	0.46 (0.47)	0.48 (0.41)	0.63 (0.48)	0.52 (0.38)	0.54 (0.31)	0.50 (0.21)	-0.62 (0.25)	0.36 (0.22)	0.48 (0.15)
Hansen test's p values	0.50	0.52	0.53	0.51	0.57	0.55	0.54	0.58	0.53	0.59
Obs.	1495	1501	1501	1501	1487	1501	1501	1492	1501	1501
Total firms	141	141	141	141	141	141	141	141	141	141

Dependent variables						
Variables	Model 11 EP_Emi	Model 12 FP	Model 13 FP	Model 14 EP_Int	Model 15 FP	Model 16 FP
Lagged of dependent variables	0.634∗∗∗ (0.001)	0.524∗∗∗ (0.003)	0.521∗∗∗ (0.002)	0.603∗∗∗ (0.004)	0.452∗∗∗ (0.007)	0.532∗∗∗ (0.002)
EPL	-0.009∗∗∗ (0.002)		0.013∗∗∗ (0.004)	-0.011∗∗∗ (0.003)		0.010∗ (0.005)
E_D	-0.018∗∗ (0.008)	0.024∗∗∗ (0.004)				
S_D				-0.032∗∗∗ (0.000)	0.015∗∗∗ (0.004)	
G_D				-0.024∗∗∗ (0.006)	0.448∗∗∗ (0.010)	
ESGD					-0.020∗∗∗ (0.002)	0.283∗∗∗ (0.007)
EP_Emi		-0.036∗∗∗ (0.009)	0.028∗∗∗ (0.002)			
EP_Int					-0.301∗∗∗ (0.006)	-0.278∗∗∗ (0.004)
F_Size	0.053∗∗∗ (0.004)	-0.043∗∗∗ (0.010)	-0.038∗∗∗ (0.001)	0.042∗∗∗ (0.006)	-0.031∗∗∗ (0.008)	0.038∗∗∗ (0.010)
C_Tech	-0.043∗∗∗ (0.003)	0.051∗∗∗ (0.010)	0.053∗∗∗ (0.005)	0.047∗∗∗ (0.002)	0.044∗∗∗ (0.003)	0.040∗∗∗ (0.008)
Lev	0.318∗∗∗ (0.034)	0.835∗∗∗ (0.026)	0.805∗∗∗ (0.027)	0.389∗∗∗ (0.002)	0.524∗∗∗ (0.012)	0.556∗∗∗ (0.014)
R&D	-0.010 (0.001)	0.032∗∗∗ (0.006)	0.030∗∗∗ (0.002)	-0.035∗∗∗ (0.001)	0.040∗∗∗ (0.008)	0.044∗∗∗ (0.013)
Polluters	0.036∗∗∗ (0.003)	0.032∗∗∗ (0.015)	0.038∗∗∗ (0.018)	0.041∗∗∗ (0.003)	0.045∗∗∗ (0.032)	0.041∗∗∗ (0.017)
Constant	-0.482∗∗∗ (0.221)	0.063 (0.223)	0.329∗∗∗ (0.261)	-0.420∗∗∗ (0.002)	0.363∗∗∗ (0.184)	0.528∗∗∗ (0.221)
Yearly effect	Yes	Yes	Yes	Yes	Yes	Yes
AR (1) p values	-2.72 (0.02)	-2.94 (0.00)	-3.16 (0.00)	-2.56 (0.01)	-3.28 (0.02)	-2.62 (0.00)
AR (2) p values	-2.59 (0.31)	0.48 (0.48)	0.61 (0.53)	2.36 (0.35)	0.48 (0.57)	0.50 (0.45)
Hansen test's p values	0.56	0.53	0.61	0.46	0.64	0.66
Obs.	1504	1501	1501	1482	1501	1501
Total firms	141	141	141	141	141	141

∗ Represents statistical significance at 5% level.

∗∗ Represents statistical significance at 10% level.

∗∗∗ Represents statistical significance at 1% level.

The second robustness check was conducted by replacing EPMs with environmental processes (EPR) as the examination of the index revealed that most of the firms have ignored implanting effective EPR. The results in Table 9 indicate that EPL has an insignificant effect on EP (models 1, 5, 8, 11, and 14), EP also represents an insignificant effect on ESGD (models 1, 5, 8, 11, and 14), and finally, EP represents an insignificant effect on FP. These findings are also compatible with the theoretical assumptions of our research implying that better EPMs lead to better EP, ESGD, and FP.

**Table 9:** Results of GMM regression (robustness checks – alternate variable EPR).

Dependent variables										
Variables	Model 1 EP_DEA	Model 2 FP	Model 3 FP	Model 4 FP	Model 5 EP_EE	Model 6 FP	Model 7 FP	Model 8 EP_PUS	Model 9 FP	Model 10 FP
Lagged of dependent variables	0.031 (0.005)	0.035 (0.010)	0.382 (0.003)	0.263 (0.008)	0.374∗ (0.002)	0.422∗∗ (0.013)	0.366∗∗ (0.001)	-0.042∗ (0.011)	0.348∗∗ (0.018)	0.242∗ (0.018)
EPL	0.010 (0.016)	0.023∗ (0.036)		0.004∗∗ (0.020)	0.046 (0.012)		0.037∗ (0.014)	0.026 (0.007)		0.024∗ (0.015)
E_D	0.048 (0.002)	0.039∗∗∗ (0.001)								
S_D					0.016 (0.006)	0.047 (0.009)				
G_D								0.054 (0.020)	0.017∗ (0.005)	
ESGD									0.028∗∗ (0.010)	0.037 (0.005)
EP_DEA			0.065 (0.003)	0.056 (0.008)						
EP_EE						0.044∗ (0.002)	0.048∗ (0.006)			
EP_PUS									-0.335 (0.010)	-0.338 (0.009)
F_Size	0.037 (0.001)	-0.029∗ (0.006)	-0.022∗ (0.002)	-0.033 (0.006)	0.021∗ (0.002)	-0.018 (0.008)	-0.0224 (0.003)	0.038 (0.008)	-0.035 (0.011)	-0.018 (0.001)
C_Tech	0.029 (0.003)	0.017 (0.007)	0.022 (0.002)	0.027 (0.013)	-0.026 (0.009)	0.041 (0.016)	0.044 (0.003)	0.042 (0.007)	0.034 (0.006)	0.038 (0.002)
Lev	0.062 (0.004)	0.060 (0.006)	0.072 (0.011)	0.063 (0.005)	-0.665 (0.006)	0.060 (0.004)	0.041∗ (0.020)	0.056 (0.003)	0.048 (0.001)	0.051 (0.004)
R&D	0.024 (0.006)	0.032∗∗ (0.001)	0.036∗∗ (0.004)	0.035 (0.008)	0.028 (0.006)	0.027 (0.003)	0.025 (0.004)	0.038 (0.009)	0.042 (0.016)	0.022∗ (0.007)
Polluters	-0.046 (0.019)	0.052∗ (0.010)	0.055 (0.010)	0.049 (0.005)	0.054∗ (0.005)	0.048 (0.007)	0.046 (0.003)	0.044 (0.011)	0.039 (0.001)	0.038∗ (0.003)
Constant	-0.462 (0.018)	0.513 (0.062)	0.604 (0.022)	0.541 (0.038)	0.493 (0.022)	-0.479 (0.014)	0.573 (0.022)	0.448 (0.038)	0.371 (0.016)	0.452∗ (0.010)
Yearly effect	Yes	Yes	Yes	Yes	Yes	Yes	Yes	Yes	Yes	Yes
AR (1) p values	-3.28 (0.02)	-3.35 (0.03)	-3.38 (0.00)	-3.47 (0.02)	-2.87 (0.03)	-2.74 (0.01)	-2.71 (0.01)	-3.19 (0.04)	-2.90 (0.03)	-2.94 (0.01)
AR (2) p values	-2.47 (0.15)	0.58 (0.37)	0.63 (0.52)	0.66 (0.40)	0.61 (0.44)	0.62 (0.20)	0.58 (0.38)	-0.57 (0.39)	0.52 (0.18)	0.42 (0.26)
Hansen test's p values	0.73	0.66	0.74	0.58	0.64	0.78	0.84	0.66	0.82	0.60
Obs.	1495	1501	1501	1501	1487	1501	1501	1492	1501	1501
Total firms	141	141	141	141	141	141	141	141	141	141

Dependent variables						
Variables	Model 11 EP_Emi	Model 12 FP	Model 13 FP	Model 14 EP_Int	Model 15 FP	Model 16 FP
Lagged of dependent variables	0.442 (0.004)	0.327 (0.008)	0.412 (0.006)	0.338 (0.005)	0.262∗ (0.017)	0.221 (0.003)
EPL	-0.320 (0.017)		0.336 (0.002)	-0.038 (0.010)		0.041 (0.008)
E_D	-0.527 (0.009)	0.042 (0.002)				
S_D				-0.021 (0.003)	0.026∗∗ (0.002)	
G_D				-0.036 (0.003)	0.039∗ (0.004)	
ESGD					-0.058 (0.007)	0.063 (0.022)
EP_Emi		-0.048 (0.002)	0.034 (0.006)			
EP_Int					-0.202∗ (0.004)	-0.359 (0.008)
F_Size	0.042 (0.007)	-0.053 (0.009)	-0.057 (0.004)	0.048 (0.002)	-0.046∗ (0.005)	0.035∗∗ (0.008)
C_Tech	-0.028∗ (0.001)	0.026 (0.003)	0.032 (0.002)	0.038 (0.000)	0.031 (0.005)	0.039 (0.004)
Lev	0.064 (0.034)	0.062 (0.008)	0.065 (0.015)	0.328∗ (0.006)	0.045 (0.002)	0.044∗ (0.011)
R&D	-0.018 (0.006)	0.026 (0.002)	0.031 (0.004)	-0.036 (0.004)	0.043 (0.004)	0.040 (0.004)
Polluters	0.069 (0.004)	0.057 (0.006)	0.056 (0.004)	0.061 (0.008)	0.056 (0.012)	0.055∗∗ (0.003)
Constant	-0.035 (0.158)	0.038 (0.196)	0.303 (0.162)	-0.023 (0.006)	0.384 (0.116)	0.415∗ (0.118)
Yearly effect	Yes	Yes	Yes	Yes	Yes	Yes
AR (1) p values	-3.14 (0.01)	-3.82 (0.02)	-3.56 (0.04)	-3.58 (0.07)	-3.46 (0.05)	-2.95 (0.10)
AR (2) p values	-3.17 (0.47)	0.38 (0.53)	0.48 (0.53)	3.20 (0.58)	0.48 (0.48)	0.40 (0.40)
Hansen test's p values	0.63	0.77	0.68	0.61	0.60	0.65
Obs.	1504	1501	1501	1482	1501	1501
Total firms	141	141	141	141	141	141

∗ Represents statistical significance at 5% level.

∗∗ Represents statistical significance at 10% level.

∗∗∗ Represents statistical significance at 1% level.

## 5 Discussion

According to H1, implementing effective EMPs have a positive effect on EP. The results of GMM regression in Table 6 (models 1, 5, and 8) confirmed that EMPs along with all control variables have a significant positive effect on EP (β = 0.004, <0.01; β = 0.07, p < 0.01; β = 0.009; p < 0.01 which confirmed that H1 is supported. The proxies used for measuring EP were EP_DEA, EP_EE, and EP_PUS indicating energy efficiency, carbon emission productivity, and carbon emission improvement, while proxies EP_Emi and EP_Int indicate carbon emission output levels (lower carbon emission levels and intensity represent positive EP and vice versa). Additionally, models 11 and 14 elucidate that firms’ EMPs have a significant and negative effect on EP which indicates a negative association between EMPs and EP (β = −0.010, p < 0.01; β = −0.002, p < 0.01. This finding established that effective EMPs have a positive effect on the EP of firms and validates the findings of past studies (see, Aslam et al., 2021; Clarkson et al., 2008; Hassan and Romilly, 2018; Moussa et al., 2020) suggested adopting good EMPs for a better EP. Additionally, this result reinforces the theoretical arguments of RBV and institutional theories that encouraged firms to adopt EMPs to reduce their environmental impacts. Hence, firms aiming to maximize their EP need to incorporate effective EMPs at operational and organizational levels (Nisar et al., 2021; Famiyeh et al., 2018; Moussa et al., 2020). However, this finding contradicts the studies of Heras-Saizarbitoria et al. (2020) and Testa et al. (2018) revealed that although EMPs are vigorously used by global firms, their effectiveness remains challenging for certain firms due to technical issues.

The effect of EP on the ESGD of firms is examined by referring to H2 which predicted that better EP has a positive effect on ESGD. The results of GMM regression (models 1, 5, 8, 11, and 14) elucidate that EP and all control variables have a significant (positive/negative) effect on ESGD (β = 0.048, p < 0.02; β = 0.065, p < 0.04; β = 0.044, p < 0.02; β = -0.023, p < 0.02; β = -0.019, p < 0.01) hence, H2 is supported. This result infers that firms aiming to improve their ESGD need to strategically improve their EP by implementing a range of environmental initiatives. This finding supports RBV's conceptual argument that requires firms to allocate strategic environmental resources to achieve sustainable growth (Hart, 1995). This result confirms the finding of recent studies (Liao, 2018; Jiang et al., 2018; Xie et al., 2019) highlighting that progressive firms may achieve a competitive advantage through their environmental initiatives leading to better ESGD. This result contradicts the findings of Miroshnychenko et al. (2017) and Yang et al. (2011) delineating that, often environmental activities incur an extra financial burden on firms therefore, firms need to carefully select their environmental initiatives.

Similarly, the effect of EP on the FP of firms is examined by referring to H3. The results (models 3 and 6) represent that EP along with all control variables has a significant positive effect on FP (β = 0.02, p < 0.01; β = 0.068, p < 0.01) hence, H3 is supported. Also, models 12 and 15 (Table 6) show that coefficient values are negative which establishes that reduction in carbon emission intensity (DEA_Int) and carbon emission levels (DEA_Emi) has a positive impact on FP and vice versa. This result validates the earlier studies (Hassan and Romilly, 2018; Aslam et al., 2021) confirming that the proxies used for measuring EP are accurate predictors of firms’ FP. While, the coefficient values (β = −0.287, p < 0.01) in model 9 are significant and negative which establishes that EP has a negative effect hence, EP_PUS proxy is not an accurate predictor of FP. Altogether, the results of H3 are consistent with the theoretical underpinnings of RBV and institutional theories validating the narrative established by earlier studies (Busch and Lewandowski, 2017; Hassan and Romilly, 2018; Jyoti and Khanna, 2021; Shahab et al., 2020) that continuous improvement in EP improves FP of firms.

To examine the mediating effect of ESGD between EP and FP, we performed the Sobel test following Baron and Kenny's (1986) technique. The results of the Sobel test examine the mediating effect of ESGD between EP and FP and are reported in Table 7. The path coefficients (β = 0.0023, p < 0.01; β = 0.0057, p < 0.01; β = -0.0019, p < 0.05; β = 0.0039, p < 0.01) are statistically significant confirming that ESGD and its three proxies (E_D, S_D, G_D) mediates the relationship between EP and FP. This finding supports H4 and endorses the arguments of past studies (Chouaibi et al., 2021; Conca et al., 2021) revealing that ESGD improves EP and FP. This result also validates RBV and institutional theories which require firms to establish certain standards and indicators to estimate EP (Kassem et al., 2017). Firms need to disclose these indicators to their influential stakeholders to facilitate their investment decisions (Chouaibi et al., 2021). This result establishes that firms may represent a better FP through public disclosure of their EMPs and EP initiatives in the form of ESG ratings which significantly help in improving their reputation and brand value (Ruan and Liu, 2021). This finding confirms the arguments of earlier studies (Di Tommaso and Thornton, 2020; Oikonomou et al., 2014) and corroborated that ESGD reduces firms' risk-taking behavior, lower operational cost, and avoids unnecessary spending on environmental activities. However, another notable feature of the mediation effect was the negative path coefficient for EP∗S_D∗FP which lays the foundation for future studies to further investigate the effect of EP on ESGD, especially the effect on social disclosure. This can be an interesting research perspective as the firms continue facing diverse social pressure due to the impact of business activities on the environment and society.

## 6 Conclusion

This study has examined the effects of environmental management practices (EMPs) on the environmental performance (EP) and financial performance (FP) of firms using environment, social, and governance disclosure (ESGD) as the mediator between EP and FP. The data between 2009 to 2020 was collected from 141 firms listed on Bursa Malaysia. The results elucidate that EMPs have a positive effect on EP. Similarly, improvements in EP positively influence the ESGD and FP of firms while ESGD scores together with its individual components mediate the relationship between EP and FP. These results are consistent with the insights of the resource-based view (RBV) and institutional theories.

Our findings delivered multiple contributions to developing literature related to optimizing corporate efficiency through environmental management. First, our results contribute to resolving environmental and economic issues of organizations through continuous improvement in environmental initiatives and integration of strategic resources. The findings have revealed that the adoption of voluntary and mandatory EMPs will allow Malaysian firms to embrace the concept of environmental legitimacy. Second, the results of the current study suggest that relative environmental programs and their disclosure improve the organizational image and brand value by reducing firms' ecological footprints. Third, the insight of this study contributes to examining the missing linkage between investments in environmental initiatives and their effectiveness. This will help the managers to justify the investments in environmental programs as the results have just confirmed that disclosure of environmental programs through ESGD will help the firms to justify investments in environmental initiatives to gain financial benefits. This will also help in achieving customer loyalty and meeting stakeholders' expectations as the firm publicly discloses its environmental, social, and governance agendas. Finally, the current study contributes to developing strategic matrices for business owners concerned about their survival in today's competitive market. The findings have suggested that investing in green programs will help in the mitigation of environmental issues and generate perpetual financial outputs.

### 6.1 Implications for practice

Our findings have several theoretical and practical implications for regulators, policymakers, and managers. The insight of this study offers a strategic roadmap for the regulators contemplating different options to reduce ecological effects and achieve environmental sustainability through zero carbon emission. Governments across the world may consider the effectiveness of EMPs in improving environmental performance which will reduce firms' negative impact on the environment by minimizing carbon emissions. The policymakers of industrial firms may use the results of this study to improve their economic situation and contribute to environmental sustainability as the findings have suggested that firms implementing a range of EMPs represent better EP and FP. The managers and business owners may use these findings to improve their firms’ reputation, image, and brand value through a toolkit established in our findings which requires a synergy between EMPs, EP, and better ESGD ratings.

### 6.2 Limitations and future studies

Like any other empirical study, the present study also has a few limitations which are deliberated briefly and future studies are recommended to address these limitations. This study uses a single country data-Malaysia hence, it is essential to remain cautious while interpreting and generalizing the results. Although the robustness of our findings is verified through statistical procedures, future studies are recommended to incorporate regional, cultural, and country-level factors as suggested by Albertini (2013) while generalizing the results. Second, the time covered in this study ranged from 2009–2020 while, the ongoing pandemic has seriously limited firms’ capabilities to implement different environmental initiatives. Future research may incorporate the recent data as it may elucidate a completely different perspective of EMPs, EP, FP, and ESGD. Third, the data analysis technique may still suffer from endogeneity problems, future studies are encouraged to consider additional measures and techniques such as difference-in-difference, generalized, two-and three-stage least squares regression to ensure the robustness of results (Xue et al., 2022). Fourth, the proxies used for estimating EP and ESGD scores may not reflect the actual picture of environmental performance and environment, social, and governance disclosure. Future studies may consider using other proxies such as water and material and ESG scores by regional rating agencies such as Thomson Reuters and MSCI to measure environmental performance and ESGD.

## Declarations

### Author contribution statement

Qaisar Ali: Analyzed and interpreted the data; Contributed reagents, materials, analysis tools or data; Wrote the paper.

Asma Salman: Conceived and designed the experiments; Performed the experiments; Analyzed and interpreted the data; Wrote the paper.

Shazia Parveen: Performed the experiments; Analyzed and interpreted the data; Wrote the paper.

### Funding statement

This research did not receive any specific grant from funding agencies in the public, commercial, or not-for-profit sectors.

### Data availability statement

Data included in article/supp. material/referenced in article.

### Declaration of interests statement

The authors declare no competing interests.

### Additional information

No additional information is available for this paper.

## Footnotes

1 The details of environmental and industrial regulations are available on the official portal of Department of Environment:https://www.doe.gov.my/portalv1/en/tentang-jas/perundangan/akta-kaedah-peraturan-arahan2/peraturan
